# Development of clinical diagnostic criteria for plaque psoriasis in children: an electronic Delphi consensus study with the International Psoriasis Council

**DOI:** 10.1111/bjd.17994

**Published:** 2019-06-28

**Authors:** E. Burden‐Teh, K. S. Thomas, S. Gran, R. Murphy

**Affiliations:** ^1^ Centre of Evidence Based Dermatology University of Nottingham Nottingham UK

Dear Editor, Psoriasis in children can be a challenging diagnosis: the clinical presentation is often more subtle, may occur in covered sites and can be an unexpected diagnosis as psoriasis is often thought to occur at older ages.^1^, ^2^ Poor recognition and delayed diagnosis of psoriasis in children can lead to inadequate treatment and lack of monitoring for comorbidities including juvenile psoriatic arthritis.^3^ Diagnostic criteria would help both clinical practice and clinical research, but to date there are none available.^4^ The aim of this study was to agree a list of expert‐derived diagnostic criteria for plaque psoriasis in children using electronic Delphi (eDelphi) consensus methodology.

The study was undertaken online in three rounds plus a feedback round (December 2015 to April 2016). The study protocol was registered on the Centre of Evidence Based Dermatology website at the University of Nottingham. The definition of consensus was decided *a priori* as being when ≥70% of participants agreed. The eDelphi follows the study design and reporting guidance by Sinha *et al*.^5^ Members of the International Psoriasis Council with an interest in paediatric psoriasis were invited to participate. As recommended by Akins *et al.,* the eDelphi aimed to include a minimum of 20 expert participants.^6^

Round 1 presented 21 clinical features identified as frequently occurring in paediatric psoriasis (< 18 years of age), based on interviews with paediatric dermatologists and a scoping review.^7^ Participants were asked to score the importance of each clinical feature in making a diagnosis on a five‐point scale (very important, important, less important, not important, not sure) and to suggest additional clinical features that they considered were missing.

Round 2 provided participants with feedback on the distribution of responses for each clinical feature. Changes were also made to the wording of some items, in‐line with participants’ feedback, and new clinical features suggested during round 1 were added. Participants were asked to re‐score the importance of each item in the light of group feedback, to score whether an item alone would support a diagnosis of psoriasis (yes, no, unsure) and to suggest options for a scoring algorithm.

Round 3 presented the list of diagnostic features that reached consensus as being important for the diagnosis of psoriasis, suggested a possible scoring algorithm to use with the diagnostic criteria and calculated percentage responses for whether an item alone would support a diagnosis. Participants were asked to re‐score the value of a single feature and vote on the scoring algorithm.

In total, 41 participants completed round 1; of these, 34 (83%) went on to complete round 2, 31 (76%) completed round 3 and 27 (66%) completed a feedback survey on the agreed criteria. Across the three rounds, most participants had over 20 years’ experience as a dermatologist (48–54%) and over 20 years’ specialist interest in psoriasis (46–48%). Most participants treated adults and children in their routine practice (56–65%). The participants represented 19 countries, and over 60% of participants were from the United States, Canada, Denmark, the Netherlands, Chile, Spain and Italy.

By the end of round 2, 16 diagnostic features reached consensus (≥ 70% agreement) as being important for the diagnosis of plaque psoriasis in children (Table 1). Three diagnostic features were identified as major criteria; that is, the presence of any of these features alone would support a diagnosis of plaque psoriasis. The remaining 13 diagnostic features were identified as minor criteria; that is, the presence of any of these features alone would not support a diagnosis of plaque psoriasis. Overall, 48% of participants felt that in the absence of at least one major criterion, three or more minor criteria would support a diagnosis of psoriasis in children (scoring algorithm). Supplementary data are available on direct application to the corresponding author.

**Table 1:** The results of round 2 and round 3 of the eDelphi consensus study presenting the group percentage scores for each diagnostic feature and categorization as major or minor criteria

Diagnostic features that reached > 70% consensus as ‘very important’ or ‘important’ and included in the consensus agreed diagnostic criteria	Total % scores for ‘very important’ and ‘important’	% agreement that a feature should be a major or minor criterion
Major criteria		
Scaly erythematous plaques on the extensor surfaces of the elbows and knees	100	93·60
Scaly erythematous plaques on the trunk triggered by a sore throat or other infection	97·10	71·90
Raindrop plaques typical of guttate disease on the trunk or limbs^a^	97·10	–b
Minor criteria		
Scale and erythema in the scalp involving the hairline	97·10	87·10
Retro‐auricular erythema (including behind the earlobes)	73·50	61·30
Scaly erythema inside the external auditory meatus^a^	73·50	63·30
Persistent well‐demarcated erythematous scaly rash anywhere on the body^a^	88·20	90·30
Fine scaly patches involving the upper thighs and buttocks	70·60	51·60
Well‐demarcated erythematous rash in the napkin area involving the crural folds	76·50	74·20
Persistent erythema in the umbilicus	88·30	60·00
Nail pitting	94·10	80·65
Onycholysis of the nail(s)	91·20	66·70
Subungual hyperkeratosis of the nail(s)	91·10	73·30
Positive family history of psoriasis	94·10	80·00
Koebner phenomenon	88·20	58·60
Fusiform swelling of a toe or a finger suggestive of dactylitis	85·30	82·10

The following diagnostic features did not reach consensus after round 2: scaly scalp; retro‐auricular skin splitting (including behind the earlobes); persistent well‐demarcated facial rash with fine or absent scale; persistent erythematous periorbital rash with fine or absent scale; well‐demarcated erythematous rash in the axilla(e); natal cleft erythema and/or skin splitting; persistent nappy rash; sleep not disturbed by itch; absence of skin xerosis. ^a^Diagnostic features suggested in the feedback from round 1 and included in round 2; ^b^59·4% agreed this item should be kept alongside ‘Scaly erythematous plaques on the trunk triggered by a sore throat or other infection’.

The strengths of the current study are that it was an international consensus study with global experts in psoriasis, who frequently treat children with psoriasis. Limitations include under‐representation of African and Asian participants. The diagnostic ability of any individual criterion and the combination of criteria that are most predictive for psoriasis are unknown. A diagnostic accuracy study is now underway to test the consensus agreed criteria and to identify the combination of features with the optimal diagnostic accuracy. This study will investigate if the major criteria have sufficient diagnostic accuracy to independently support a diagnosis of psoriasis, and whether criteria that overlap with other skin diseases (i.e. are not predictive of psoriasis) need to be removed.

This eDelphi consensus study provides a list of expert agreed diagnostic features and is the first step in developing diagnostic criteria for plaque psoriasis in children.
